# Structure and mechanism of the formation of core–shell nanoparticles obtained through a one-step gas-phase synthesis by electron beam evaporation

**DOI:** 10.3762/bjnano.6.89

**Published:** 2015-03-31

**Authors:** Andrey V Nomoev, Sergey P Bardakhanov, Makoto Schreiber, Dashima G Bazarova, Nikolai A Romanov, Boris B Baldanov, Bair R Radnaev, Viacheslav V Syzrantsev

**Affiliations:** 1 Department of Physics and Engineering, Buryat State University, Smolina street 24a, Ulan-Ude, 670000, Russia; 2 Institute of Physical Materials Science, Siberian Branch of the Russian Academy of Sciences, Sakhyanova str. 6, Ulan-Ude, 670047, Russia; 3 Institute of Theoretical and Applied Mechanics, Siberian Branch of the Russian Academy of Sciences, Institutskaya str. 4/1, Novosibirsk, 630090, Russia; 4 Department of Physics, Novosibirsk State University, Pirogova street 2, Novosibirsk, 630090, Russia

**Keywords:** core–shell, electron beam evaporation, gas phase, mechanism of formation, one-step

## Abstract

The structure of core–shell Cu@silica and Ag@Si nanoparticles obtained in one-step through evaporation of elemental precursors by a high-powered electron beam are investigated. The structure of the core and shell of the particles are investigated in order to elucidate their mechanisms of formation and factors affecting the synthesis. It is proposed that the formation of Cu@silica particles is mainly driven by surface tension differences between Cu and Si while the formation of Ag@Si particles is mainly driven by differences in the vapour concentration of the two components.

## Introduction

Core–shell type nanoparticles are a type of biphasic materials which have an inner core structure and an outer shell made of different components. These particles have been of interest as they can exhibit unique properties arising from the combination of core and shell material, geometry, and design [1–2]. Additionally, they have been designed so that the shell material can improve the reactivity, thermal stability, or oxidative stability of the core material or to use an inexpensive core material to carry a thin, more-expensive shell material [1–2]. Thus, they have found wide applicability in fields such as biomedicine, electrical and semiconducting materials, and catalysts [1–2].

The majority of core–shell particles are synthesised using solution methods and usually involve two steps: synthesis of the core structure followed by coating the core structure with the shell material. Gas-phase synthesis techniques exist and usually involve chemical vapour deposition (CVD) or pulsed laser deposition (PLD) [1–2]. However these techniques also involve multiple steps, usually depositing the shell material onto an already formed core structure, and use substrates [3–5]. For these established techniques, the mechanisms of formation have been investigated and the various parameters which affect the particle formation are known [1,6]. Recently, the authors have synthesized core–shell Ag–Si and Cu–Si type particles in a new way using electron beam evaporation [7–8]. In this method, the core–shell particles are synthesized in one-step directly from the gas phase without substrates. The precursors used are elemental materials rather than chemical compounds which are degraded into the final core or shell materials. Thus, this method may also be a more economical method of synthesising certain types of core–shell particles. Previous nanopowders synthesized with this method have been produced at kg/h rates [9–11]. As single-step synthesis of core–shell materials from elemental gas phase precursors has, to the authors’ knowledge, never been performed before, the mechanism of formation and the factors influencing the synthesis are unknown. Thus, the current work attempts to use data from the two synthesized Ag@Si and Cu@silica particles to elucidate the mechanism by which the core–shell nanoparticles may form. As the electron beam evaporation method is a high-energy method, the reaction cannot be observed as it occurs. Thus, the resultant particles are characterized in-depth in order to illuminate how they formed. This work can aid future researchers hoping to produce new types of core–shell particles using the electron beam evaporation method.

## Results and Discussion

### Structure of Cu@silica particles

**Figure 1:**
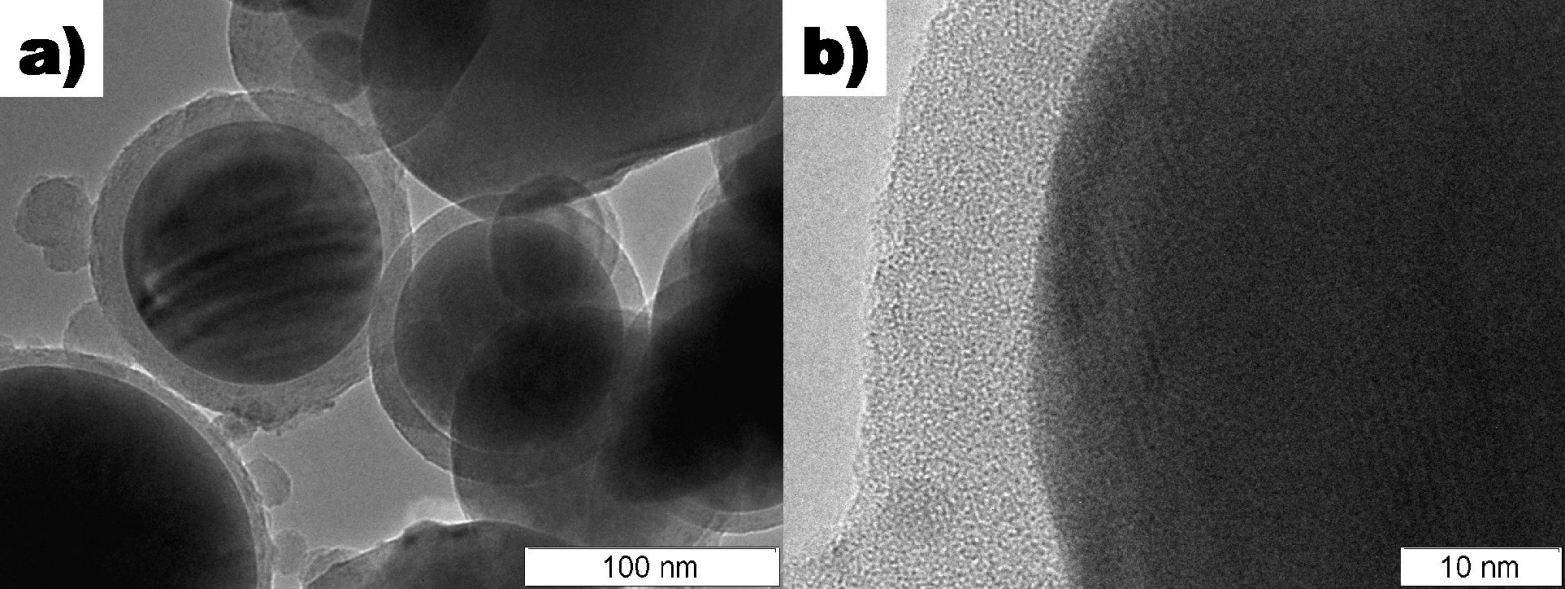
TEM micrograph of the Cu@silica nanoparticles. a) Overview, b) detail of the core and shell structure.

**Figure 2:**
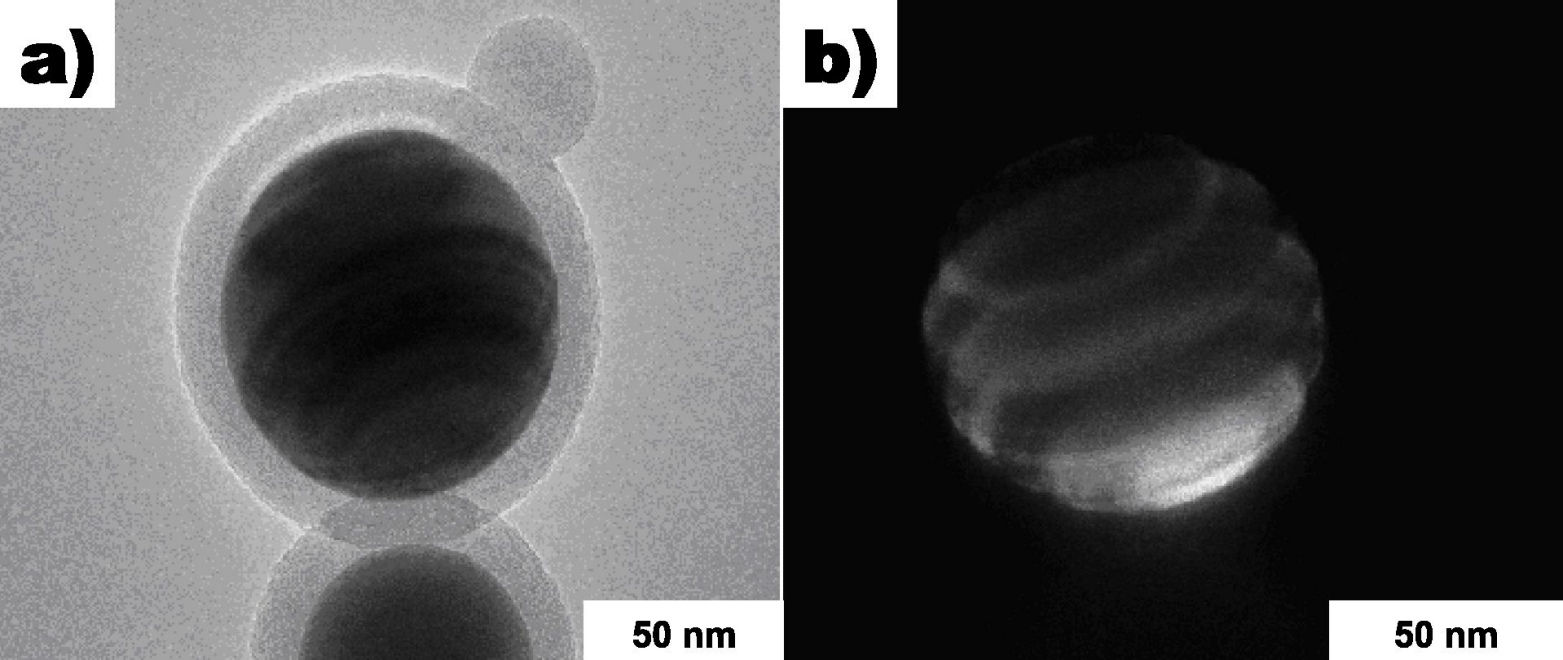
TEM micrograph of a Cu@silica nanoparticle. a) Bright field, b) dark field.

In the original work [7], XRD analysis on the bulk Cu@silica samples revealed Cu, Cu_2_O, and CuO phases in the powder. A TEM micrograph of the synthesized Cu@silica nanoparticles is shown in Figure 1a. The average particle size was 119 ± 73 nm. A higher magnification image of the shell and core structure is shown in Figure 1b. In Figure 1b, it can clearly be seen that the shell structure is amorphous and much less dense than the core structure and some crystal planes are clearly visible in the core structure. SAED measurements were performed on various areas of the core structure and throughout the core, Cu(111) was measured. The core structure was also measured after 5 years of storing the particles at atmospheric conditions in a non-airtight container and the core structure was unchanged; indicating that the shell structure is very effective at preventing contact of the Cu core with oxygen. An XRD of the Cu@silica powders from a different batch which contained less copper oxides is shown in Supporting Information File 1, Figure S1. Some particle cores, when observed with the correct focus and electron beam strength exhibited ring structures indicative of multiple contact twinning as shown clearly in Figure 2 (also seem in one particle in Figure 1a). This reveals that the core structure consists of domains of monocrystals rather than randomly oriented polycrystalline grains or a single monocrystal.

The shell thickness was about 11 nm for most particles. As the shell is amorphous, it was not possible to measure its crystallographic structure. There were also a few small particles not connected to a core which had the same contrast as the shell material on the TEM. When EDX measurements of the core–shell particles were measured, Cu, Si, and O were measured. EDX measurements performed on the “unbound” shell material measured Si and O. The relative atomic composition of the shell material was about 64% Si and 36% O. Thus, the shell is likely a mixture of amorphous Si, SiO, and SiO_2_. As the argon atmosphere in which the particles were synthesized is considered quite pure, the oxidation of the shell for the Cu@silica particles but lack of it in the Ag@Si particles suggests that the majority of the oxygen likely originated from the starting copper ingot which is known to contain some oxygen. As Si has a higher affinity for oxygen than Cu, oxygen released by the evaporation of the copper or dissolved in the copper would be taken up by the silicon during particle formation. From HRTEM images, it is clear that the shells are not completely spherical and have some roughness (lumps) in their structure. This may result from either small solid Si particles agglomerating onto the core, onto an already formed shell, or disturbances to the shell as it was forming by the surrounding environment. Some clumps of different contrast were also observed on the surface of some shells and were identified to be CuO and Cu_2_O by SAED measurements. Thus, after formation of the shell, some copper had also adsorbed onto the shell surface. These were likely oxidized after the nanoparticles were exposed to the air.

**Figure 3:**
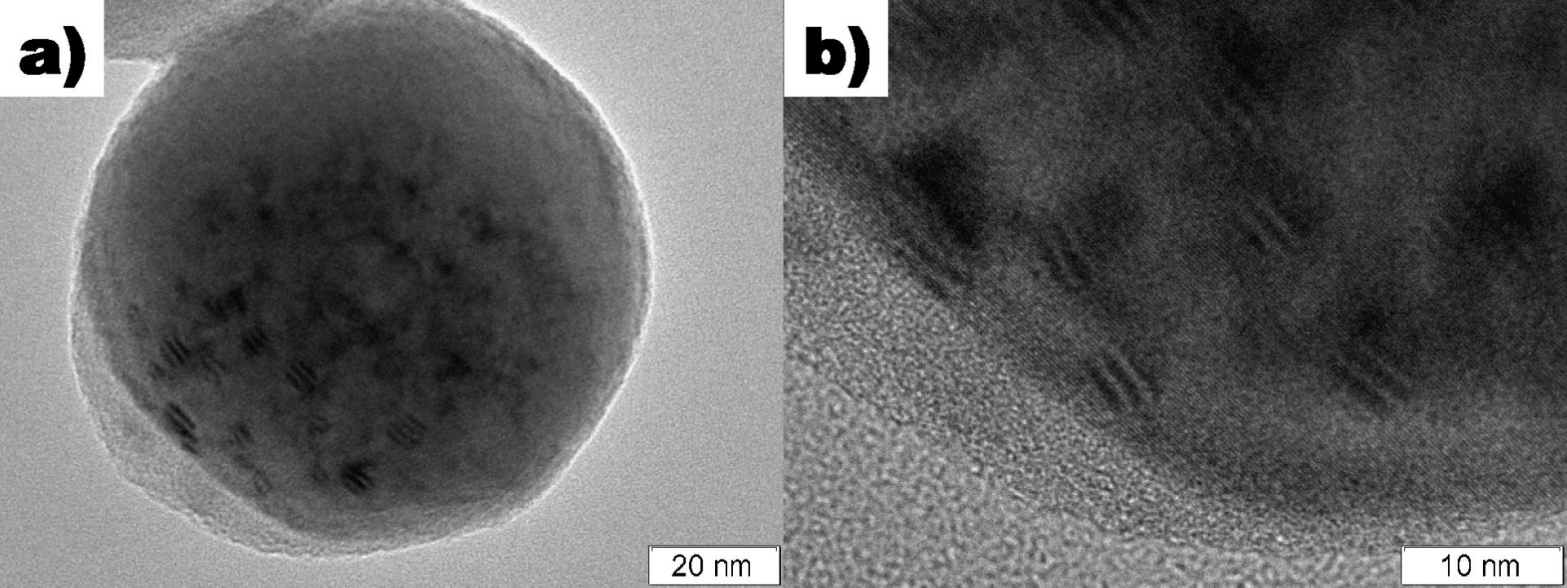
TEM micrograph of a Cu particle with an incomplete shell demonstrating moiré patterns where Cu_2_O has grown on the Cu. a) View of whole particle, b) detail of the moiré patterns.

In addition to the core–shell particles, a few particles were observed with very uneven or possibly incomplete shells (Figure 3). These particles had spots with what appeared to be moiré patterns; interference patterns which form due to differences in the lattice orientation between two crystal planes. Moiré patterns can occur due to two identical planes being rotated with respect to one another, two parallel planes with dissimilar spacings, or a combination of the two. SAED measurements on these regions showed the presence of Cu(111), Cu_2_O(111), and a moiré spacing of 1.3100 nm. The moiré spacing, *D*, between two phases can be calculated by

[1]
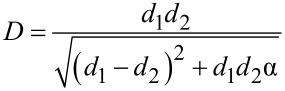


where *d*_1_ and *d*_2_ are the interplanar spacings of the two phases and α is the angle between the two lattices [12]. Fitting the measured moiré spacing to the moiré spacing calculated based on the measured d-spacings, a relative angle of α = 0.093° was fitted. Thus, the upper layer is essentially parallel to the bottom plane. It is known that the minimal value of the crystal formation and surface energy is obtained when a crystal plane grows on a substrate of the same crystal structure. As the crystal structure of both of Cu and Cu_2_O are cubic and the moiré patterns are caused by parallel planes, this suggests that Cu_2_O(111) grew epitaxially onto the exposed Cu(111). Although it can not be determined definitively from the 2-dimentional TEM images, it may be possible that the Cu_2_O grew in holes in the shell. It is unknown when the Cu_2_O formed – during or after the experiment but as the rest of the core structure was not further oxidized, the “patching” of the holes may have protected the core from further oxidation. As only (111) planes were measured on the moiré patterns, the multiple contact twinned structure of the core is further supported.

### Structure of Ag@Si particles

**Figure 4:**
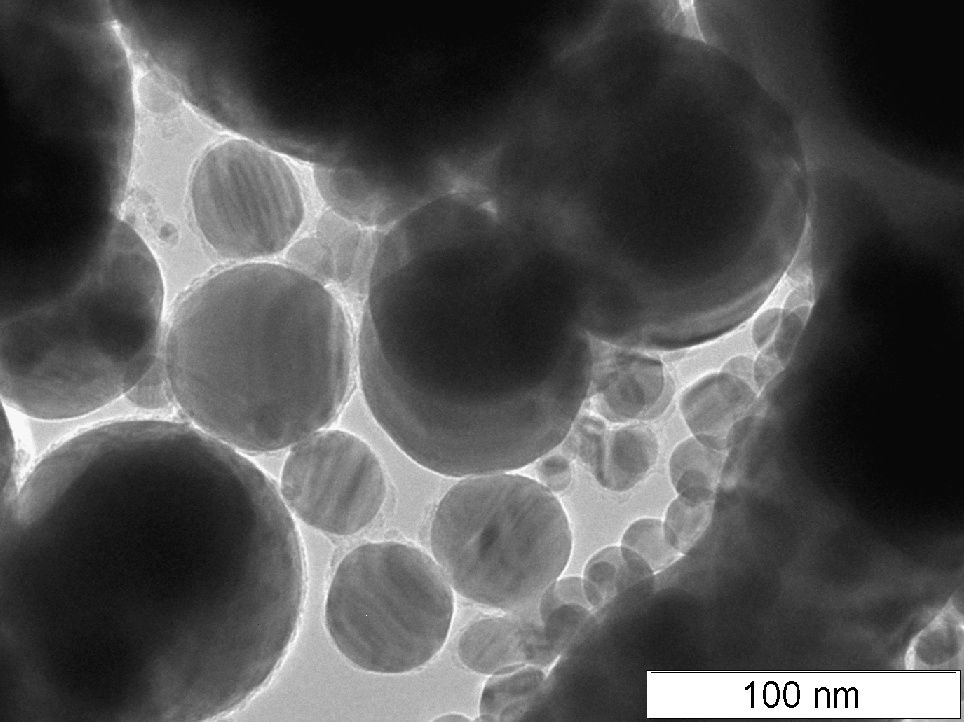
TEM micrograph of the Ag@Si nanoparticles along with Ag agglomerates.

An X-ray diffractogram of the Ag@Si particles is shown in Supporting Information File 1, Figure S2, showing only crystalline silver. A TEM image of the synthesized Ag@Si particles is shown in Figure 4. In general, the powder synthesized from the Ag–Si system contained a lower yield of Ag@Si spherical core–shell particles compared to the Cu–Si system and more interconnected and deformed silver agglomerates. Additionally, in general, the shell structures were much thinner and more uneven on the Ag@Si particles compared to the Cu@silica particles. Thus, this indicates a lower affinity of the shell for the core. The core was confirmed to be crystalline Ag with no intermixing of Si and the shell was still amorphous Si. In some Ag@Si particles, interesting patterns were observed but they were not as regular as on the Cu@silica particles and were not thought to be due to twinning defects. The cause of these patterns is still unknown. The average particle size in the Ag@Si system is 11 ± 10 nm, much smaller than the Cu@silica particle sizes.

### Formation of Cu@silica particles

According to calculations in [13], the main reasons multi-component particles form into core–shell structures is either a drive to decrease the surface energy of the system or differences in the atomic sizes of the component materials.

In the case of the Cu–Si system, the difference in covalent radius between Cu (132 pm) and Si (111 pm) is insignificant and thus size effects are not considered relevant for this system. In many cases, the surface tension of a liquid has a temperature dependence of the form

[2]
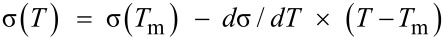


which is valid for a certain temperature range above the melting temperature (*T*_m_) of the material where σ(*T*_m_) is the surface tension at the melting point of the material and *d*σ/*dT* is the rate of change of the surface tension with temperature [14]. The empirical dependence of the surface tension of copper with temperature is

[3]
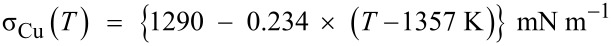


[15]. The surface tension of silicon varies as

[4]
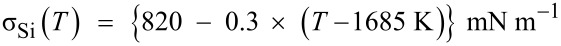


**Figure 5:**
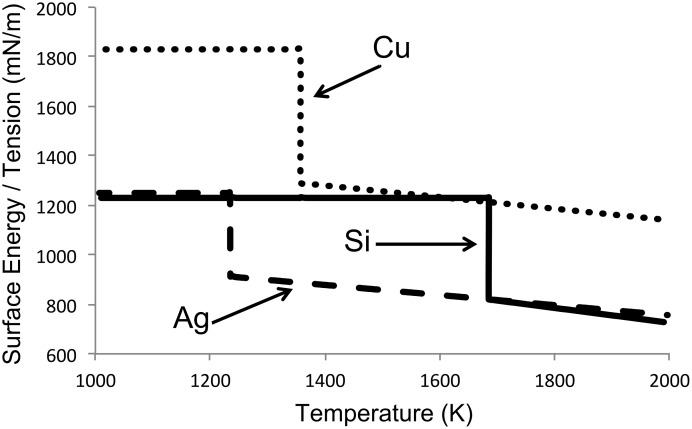
Graph of the dependence of the surface tension of Si, Cu, and Ag with temperature.

[16]. Below the melting point, when the materials are solid, the surface energy is the solid equivalent of the surface tension. The surface energy values for Cu and Si are 1830 and 1230 mN m^−1^, respectively [17]. The dependence of the surface tension and energy of Si, Cu, and Ag with temperature are plotted in Figure 5. As can be seen, at all temperatures, the surface tension of Cu is significantly higher than that of Si. Thus, reduction of the surface energy of the particles by coating Cu with Si is likely to be the main driving force for making the Si–Cu vapour condense into Cu@silica particles.

A mechanism of formation of Cu@silica particles can be proposed considering that both the melting and boiling temperatures of Si are higher than that of Cu (*T*_b_(Si) = 3538 K, *T*_b_(Cu) = 2835 K) as is the surface tension. It is reasonable to assume that as the mixed vapour cools, droplets of Cu and Si condense from the gas phase and come into contact. In the aggregated droplets, the silicon atoms will segregate to the surface of the droplet due to the difference in surface tension. During this segregation process, the silicon will react with any oxygen dissolved inside the droplet or in the atmosphere to form SiO_2_ as Si has a higher affinity for oxygen than Cu. The silicon on the surface will then solidify long before the inner copper to form the shell; the amorphous nature resulting from the SiO_2_. This solid shell around the liquid copper allows the Cu to crystallize undisturbed from the outer atmosphere; forming multiple contact twinned structure instead of polycrystals. Excess Si and Cu which did not form into mixed droplets may later agglomerate onto the shell surface; the adsorbed copper oxidizing when the powder is exposed to air. Particles with moiré patterns may form when insufficient Si droplets mix with Cu; leaving uncovered areas on the copper that could be oxidized.

The variations in the particle sizes of the Cu@silica particles are thought to be due to local fluctuations in the vapour concentration of Cu and Si. Fluctuations in local vapour concentrations can easily occur from the carrier gas flow and convection currents in the Cu–Si liquid being evaporated. As the thickness of the silica shell is relatively constant compared to the particle sizes, this suggests that the silica shell stops the growth of the core Cu. Thus areas containing higher Si concentrations would form smaller core–shell particles while in areas with lower Si concentrations, the Cu core would grow larger before the growth was halted by the shell formation.

### Formation of Ag@Si particles

In the case of the Ag–Si system, the atomic radii (*r*_Ag_ = 145 pm and *r*_Si_ = 111 pm) are also not significantly different. As seen from Figure 5 (using

[5]
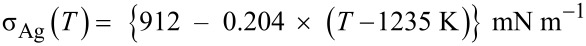


[18] and a surface energy of Ag of 1250 mN m^−1^ [17]), the surface tension of Ag and Si are extremely close. Thus, neither atomic size effects nor surface tension effects are likely to be responsible for the formation of core–shell structures.

In initial experiments with the Ag–Si system, the beam current was varied for the evaporation of the target. It was found that core–shell particles were only obtained at a beam current of 4 mA. At higher beam currents of 5 and 7 mA, core–shell nanoparticles were not created but instead large Ag particles with small Si particles adsorbed onto them. Therefore, it seems likely that the relative concentration of Ag and Si in the evaporated vapour played a key role in the formation of core–shell structures. As the vapour pressure of Ag (100 kPa at 2433 K [19]) is higher than that of Si (100 kPa at 3537 K [19]), at higher beam currents, the Si:Ag vapour ratio would decrease. Only at the beam current of 4 mA was the Si content in the vapour sufficient to create the core–shell particles. Due to the relative thinness of the Si shell compared to the size of the core structure, the lack of free Si particles found in the sample, and the high number of deformed or interconnected Ag particles, it is thought that the vapour in the reported experiment still contained a higher portion of Ag compared to Si. The ratio of Si to Ag was also varied and core–shell particles only formed with higher Si to Ag ratios; supporting the need for sufficient Si in the vapour phase for core–shell structure formation. The carrier gas flow rate was varied as well but mainly affected the particle sizes, with smaller particles forming with higher flow rates due to faster cooling of the vapour.

Based on this information, the following mechanism of formation for the Ag@Si particles is proposed: Initially, particles of Si and Ag condense from the vapour. As the Si concentration is low in the vapour, only very small amorphous Si particles form while larger crystalline Ag particles are able to form. No intermixing occurs as Si has an extremely low solubility in Ag (the maximum solubility being 4 × 10^–4^ at % at 1623 K [20]). Due to van der Waal interactions, the particles agglomerate. As the relative size of the Ag particles are much larger compared to the Si particles, the Si tends to agglomerate onto the surface of the Ag particles. However, as the Si particle concentration is low, many Ag particles are not fully covered and thus uncovered areas of the Ag agglomerate with other exposed Ag on other particles.

The size distribution of the Ag@Si particles was much smaller than that of the Cu@silica particles. However, for the Ag–Si system many larger Ag agglomerates were found along with the core–shell particles. Due to the limited amount of Si, only the smaller Ag particles could be coated fully by the Si to form complete core–shell particles; narrowing the Ag@Si size distribution.

## Conclusion

The structure and mechanism of formation of Cu@silica and Ag@Si core–shell nanoparticles synthesized using high-powered electron beam evaporation were investigated. While the mechanism of formation differ between the systems, in general, the main factors causing core–shell structure formation are the relative vapour concentration of the materials, surface tension differences, and differences in melting temperature of the component materials. Besides changing the precursor materials, the main experimental parameters that can affect the formation of core–shell particles is the electron beam strength. The formation of the Cu@silica core–shell nanoparticles is thought to predominantly be driven by surface tension differences between the core and shell material at the melting temperature of the shell. In this mechanism, it was important that the shell material solidify before the core material to create these structures. In the case of the Ag@Si system, the relative concentration of the two elements in the vapour was thought to be the most important parameter; the low concentration Si thinly adsorbing onto the surface of larger Ag particles. These results may guide the synthesis of future core–shell type particles through the electron-beam evaporation method.

## Experimental

The principle of operation of the experimental setup for producing the Cu@silica and Ag@Si composite nanoparticles and schematics of the device are given in [7–8]. Briefly, an ELV-6 industrial electron accelerator was used to produce an electron beam to evaporate the chosen materials. The accelerator allows the electron beam to be released into a non-vacuum environment with an electron energy of 1.4 MeV, beam current of up to 75 mA, and beam power density of up to 5 × 10^6^ W/cm^2^. A crucible was filled with the materials to be evaporated. For the synthesis of Cu@silica particles, Si and Cu were placed in a graphite crucible with a weight ratio of 20:1 with Cu on the bottom. The Ag@Si particles were synthesized with a Si:Ag weight ratio of 10:1 with Ag on the bottom. The materials were irradiated by the electron beam first, at a low current to melt the materials, then at a higher current to evaporate them. The heating rate could reach as much as 1000 K/min and the surface temperature of the melt could reach 5000 K. Throughout the experiment, Ar gas was flowed through the evaporation chamber and carried the evaporation gasses to a condensation chamber where the nanoparticles were formed and then through a filter from which the nanoparticles were collected.

The characterization of the nanoparticles was carried out by transmission electron microscopy (TEM), high-resolution TEM (HRTEM), selective area electron diffraction (SAED), and energy dispersive X-ray fluorescence (EDX) analysis. These measurements were performed on a JEM-2010 TEM (JEOL, Japan, 200 kV accelerating voltage, 0.14 nm resolution) equipped with an EDX (EDAX Co.) spectrometer (130 eV energy resolution, 1 nm spatial resolution). To perform the measurements, the core–shell nanopowders were diluted in ethanol, subjected to ultrasonic dispersion, and precipitated onto a carbon film fixed to a copper grid.

## Supporting Information

File 1 XRD analysis.
