# Supplementary material for: Structure and mechanism of the formation of core–shell nanoparticles obtained through a one-step gas-phase synthesis by electron beam evaporation
Source: Beilstein J Nanotechnol. 2015 Mar 31;6:874–80. doi: 10.3762/bjnano.6.89 (PMC4419578; doi:10.3762/bjnano.6.89)
Supplement: File 1 — XRD analysis. [file Beilstein_J_Nanotechnol-06-874-s001.pdf]

Supporting Information

for

Structure and mechanism of formation of core–shell nanoparticles obtained through one-step gas-phase synthesis by electron beam evaporation

Andrey V. Nomoev[1,2,*], Sergey P. Bardakhanov[1,3,4], Makoto Schreiber[1], Dashima G. Bazarova[1], Nikolai A. Romanov[1], Boris B. Baldanov[1], Bair R. Radnaev[1] and Viacheslav V. Syzrantsev[3]

Address: [1]Department of Physics and Engineering, Buryat State University, Smolina street 24a, Ulan-Ude, 670000, Russia, [2]Institute of Physical Materials Science, Siberian Branch of the Russian Academy of Sciences, Sakhyanova str. 6, Ulan-Ude, 670047, Russia, [3]Institute of Theoretical and Applied Mechanics, Siberian Branch of the Russian Academy of Sciences, Institutskaya str. 4/1, Novosibirsk, 630090, Russia and [4]Department of Physics, Novosibirsk State University, Pirogova street 2, Novosibirsk, 630090, Russia

Email: Andrey Nomoev - nomoevav@mail.ru

* Corresponding author

XRD analysis

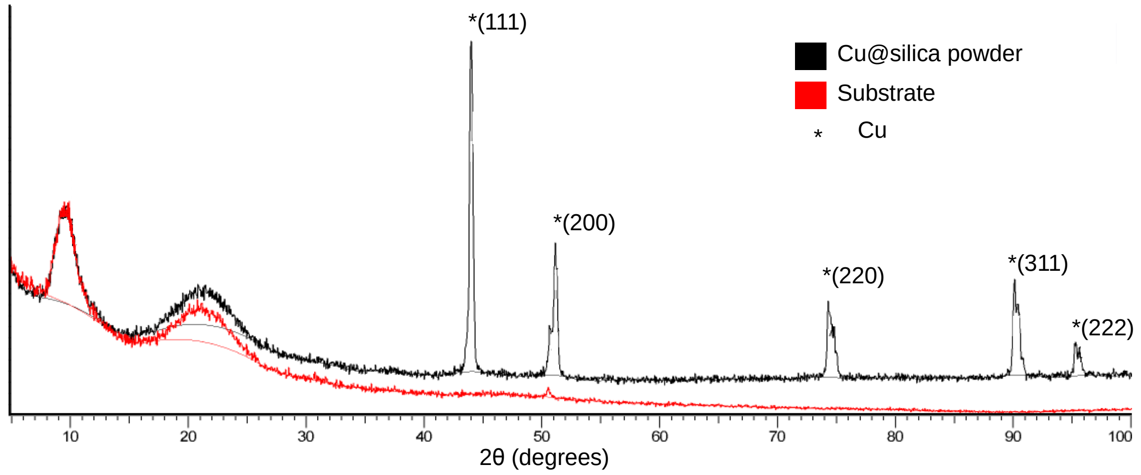

Figure S1: XRD of the obtained Cu@silica powders (Bruker D2 Phaser, Cu K_{α} λ = 1.5218 Å).

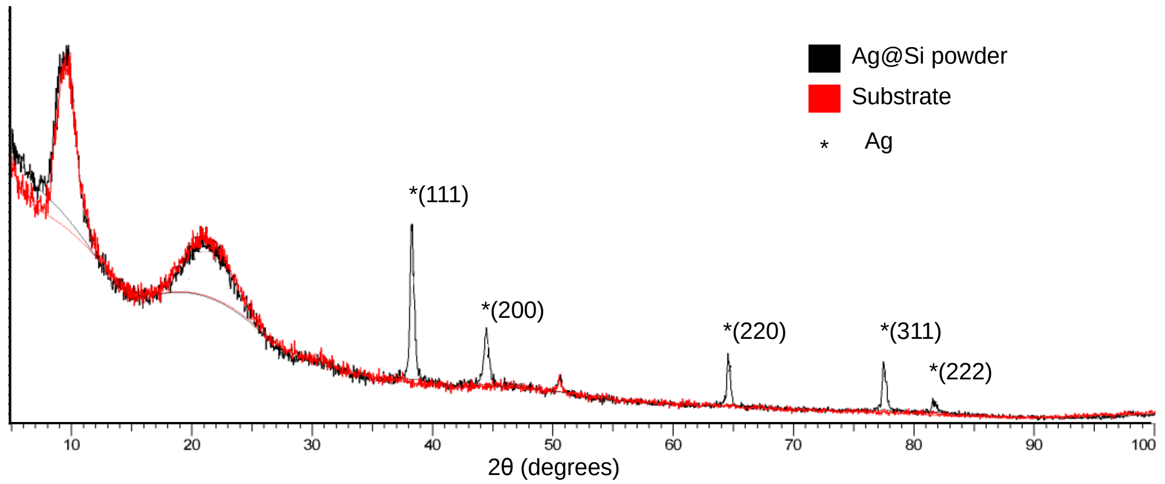

Figure S2: XRD of the obtained Ag@Si powders (Bruker D2 Phaser, Cu K_{α} λ = 1.5218 Å).
